# Ability of *Saccharomyces cerevisiae* MC87-46 to assimilate isomaltose and its effects on sake taste

**DOI:** 10.1038/s41598-019-50384-w

**Published:** 2019-09-26

**Authors:** Seitaro Tsutsumi, Mai Mochizuki, Kiyota Sakai, Akane Ieda, Reiji Ohara, Shun Mitsui, Akitoshi Ito, Tatsuya Hirano, Motoyuki Shimizu, Masashi Kato

**Affiliations:** 1 Faculty of Agriculture, Meijo University, 1–501, Shiogamaguchi, Tenpaku-ku, Nagoya, Aichi 468-8502 Japan; 2 Food Research Centre, Aichi Centre for Industry and Science Technology, 2-1-1 Shimpukuji-cho, Nishi-ku, Nagoya, Aichi 451-0083 Japan

**Keywords:** Applied microbiology, Cellular microbiology

## Abstract

Recently, wild strains of *Saccharomyces cerevisiae* isolated from a variety of natural resources have been used to make bread, beer, wine, and sake. In the current study, we isolated wild *S. cerevisiae* MC strain from the carnation (*Dianthus caryophyllus L*) flower and produced sake using its cerulenin-resistant mutant strain MC87-46. Then, we characterized the components, including ethanol, amino acids, organic acids, and sugars, in the fermented sake. Sake brewed with MC87-46 is sweet owing to the high content of isomaltose, which was at a concentration of 44.3 mM. The low sake meter value of −19.6 is most likely due to this high isomaltose concentration. The genomic DNA of MC87-46 encodes for isomaltases *IMA1*, *IMA2*, *IMA3, IMA4* and *IMA5*, as well as the isomaltose transporter gene, *AGT1*. However, these genes were not induced in MC87-46 by isomaltose, and the strain did not possess isomaltase activity. These results show that MC87-46 cannot utilize isomaltose, resulting in its accumulation in the fermented sake. Isomaltose concentrations in sake brewed with MC87-46 were 24.6-fold more than in commercial sake. These findings suggest that MC87-46 may be useful for commercial application in Japanese sake production because of its unique flavour and nutrient profile.

## Introduction

Japanese sake is a traditional rice wine beverage. During the process of brewing sake, the starch in rice is saccharified by various enzymes produced by the koji mold, *Aspergillus oryzae*, and the resultant glucose is fermented to ethanol by the sake yeast, *Saccharomyces cerevisiae*^1^. This fermentation process proceeds without accumulation of high levels of sugars and generates various low molecular weight compounds that constitute the complex flavour of sake^2–8^. These compounds are present at different concentrations in various sakes^2,5,7^.

The taste of sake is affected by the sake meter value, balance of organic acids and esters, and various sugars. Sake contains more sugars than other alcoholic beverages and the majority of the sugar in commercial sake is glucose, which is thought to be the cause of the sweetness of sake^9^. Disaccharides, such as maltose, isomaltose, nigerose, and kojibiose have been reported to be present in sake at levels reaching 2,000–8,000 ppm^10–12^. The total amount of trisaccharides, including maltotriose, isomaltotriose, panose, and isomaltotetraose is estimated to be 2,000–5,300 ppm^13,14^. These oligosaccharides are produced from starch by various enzymes secreted from *A. oryzae*. Disaccharides, such as isomaltose, kojibiose, and nigerose are generated by the transglycosylation activity of α-glucosidase produced during sake brewing^15–17^. These resulting oligosaccharides are then converted to ethanol by the yeast, *S. cerevisiae*^4^.

*S. cerevisiae* is a domesticated species that has been associated with the production of several alcoholic beverages^3,5,6^. In many instances, yeast strains are specialized for the production of sake^4^, wine^18,19^, beer^19^, and bread^19^. This has led to the common view that several alcoholic beverages produced by the same yeast have similar characteristics. Recently, wild strains of *S. cerevisiae* isolated from a variety of natural resources were used to make bread, beer, wine, and sake. Studies of wild *S. cerevisiae* strains revealed variations in their production of wine aroma and flavour metabolites^20–22^. The use of different yeasts isolated from various natural resources also affects the flavour profile^20–22^.

In the current study, we isolated a wild *S. cerevisiae* strain from the carnation flower (*Dianthus caryophyllus L*) grown on a farm at Meijo University and subsequently introduced cerulenin-resistance and then used the modified strain to brew sake. Furthermore, we determined the components in the fermented sake, including ethanol, amino acids, organic acids, and sugars. We also analysed the sugar assimilation properties of the yeast strain.

## Results

### Isolation and identification of wild *S. cerevisiae* for small-scale sake brewing

Wild *S. cerevisiae* strains were isolated from carnation flowers grown in the experimental farm at the Meijo University. Yeasts were identified by sequencing the ITS1/ITS2 region of the *18S rRNA* gene (data not shown), and then a wild *S. cerevisiae* MC strain was selected. After EMS mutagenesis, we selected nine cerulenin-resistant strains for improving the ethyl caproate production ability^23,24^, and used them to brew sake. Among them, one strain, MC87, that possessed the highest ethyl caproate productivity was selected and used for subsequent experiments (Fig. S1). Compared with a MC strain, MC87 produced 7.3-fold more ethyl caproate (Fig. S1). Subsequently, MC87 strain was suspended in 20% (v/v) ethanol for improving ethanol tolerance, based on an adaptive laboratory evolution method, as previously described^25^. We isolated an ethanol-resistant strain, MC87-46 with higher ethanol tolerance than the MC87 strain (Fig. S2). However, MC87-46 showed lower ethanol tolerance than the standard *S. cerevisiae* strain, Kyokai No. 9 (K901) strain^26^.

**Figure 1:**
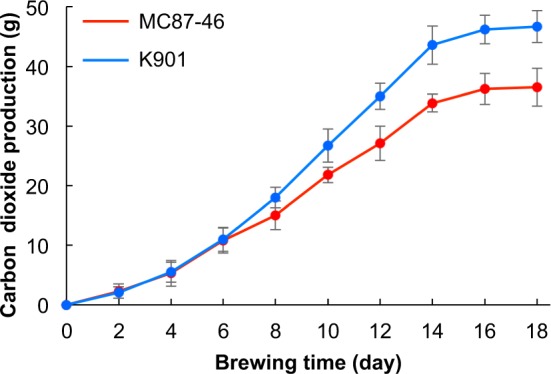
Time course of CO_2_ production in sake fermented by MC87-46 and K901. Small-scale sake brewing with either *S. cerevisiae* MC87-46 or K901 strain was carried out at 15 °C for 18 days. Data are presented as mean values ± standard deviation (error bars) of three independent experiments.

We prepared sake with the MC87-46 strain, as well as K901 strain and determined the amount of carbon dioxide produced during the fermentation process (Fig. 1). MC87-46 produced 19% less carbon dioxide than K901 (Fig. 1). This is consistent with the lower levels of ethanol and lower sake meter values of sake brewed with MC87-46 compared with those of sake brewed with K901 (Table 1). The acidity and amino acid levels in the sake produced by MC87-46 were 1.4- and 1.3-fold higher, respectively, than those in the sake produced by K901. This implied that sake fermented by MC87-46 would be sweeter and sourer than sake fermented by K901.

**Table 1:** Profiles of sake brewed with K901 and MC87-46.

	Ethanol (%)	Sake meter	Acidity	Amino acidity
K901	20.2 ± 3.2	9.9 ± 1.7	3.7 ± 0.72	1.5 ± 0.39
MC87-46	16.4 ± 2.9	−19.6 ± 1.5*	5.1 ± 0.67	1.9 ± 0.45
	**Organic acid concentrations (mM)**			
	**Malic acid**	**Succinic acid**	**Lactic acid**	**Acetic acid**
K901	1.7 ± 0.21	5.7 ± 0.83	3.4 ± 0.57	7.3 ± 1.7
MC87-46	1.4 ± 0.36	6.0 ± 1.1	3.4 ± 0.63	13.8 ± 3.1*
	**Aroma component concentrations (ppm)**			
	**Ethyl acetate**	**Ethyl caproate**	**Isoamyl acetate**	**Isoamyl alcohol**
K901	5.7 ± 0.6	1.9 ± 0.4	8.1 ± 0.2	231.0 ± 8.4
MC87-46	2.0 ± 0.4*	1.6 ± 0.3	2.2 ± 0.1*	223.6 ± 8.0

Small-scale sake was brewed for 18 days at 15 °C. Data are presented as mean values ± standard deviation (error bars) of three independent experiments. *p < 0.01, Student’s t test.

### Quantification of organic acids and aroma components in fermented sake

The concentrations of the major organic acids in the sakes were quantified using HPLC (Table 1). Malic acid, succinic acid, lactic acid, and acetic acid were detected in sake produced by both strains. Among them, only acetic acid was increased 1.9-fold in sake fermented with MC87-46 than in that fermented with K901 (Table 1). This suggests that acetic acid was involved in the acidity level of sake brewed with MC87-46.

The aroma components in the sakes were also quantified using headspace gas chromatography. Isoamyl alcohol and ethyl caproate produced by MC87-46 were similar to those produced by K901 (Table 1). The isoamyl acetate and ethyl acetate levels in the sake produced by K901 were 3.70- and 2.85-fold higher, respectively, than those in the sake produced by MC87-46 (Table 1). Compared with the wild *S. cerevisiae* MC strain isolated from the carnation flowers, MC87-46 produced 7.0-fold more ethyl caproate (Fig. S1). In addition, a mutation in *FAS2* gene at 3748 position was identified by DNA sequencing (data not shown). These results indicated that the ethyl caproate production ability of MC87-46 was improved by selecting for the cerulenin-resistant colony after EMS mutagenesis.

### Quantification of sugars in fermented sake

**Figure 2:**
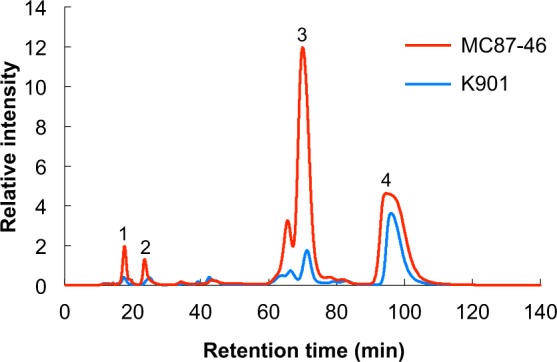
HPLC analysis of sugars in sake fermented by MC87-46 and K901. Sugars in sake fermented by either MC87-46 or K901 at 15 °C for 18 days were quantified using a reducing-sugar HPLC analytical system. *Peak* 1, panose; *peak* 2, unknown; *peak* 3, isomaltose; *peak* 4, glucose.

**Figure 3:**
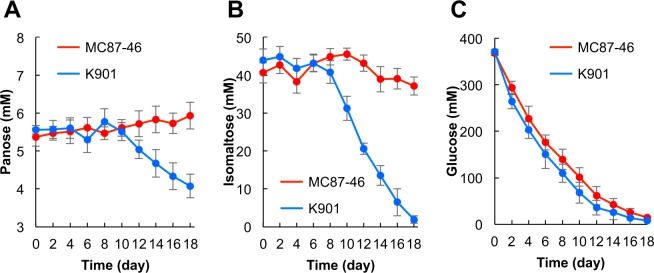
Time course of sugar concentration during the fermentation process. Concentrations of panose (**A**), isomaltose (**B**) and glucose (**C**) in the fermentation process by MC87-46 and K901 strains. Data are presented as mean values ± standard deviation (error bars) of four independent experiments.

Sugars in sake fermented by both MC87-46 and K901 strains were identified by thin-layer chromatography (TLC) and gas chromatography-mass spectroscopy (GC-MS) (Fig. S3), and quantified using a reducing-sugar high-performance liquid chromatography (HPLC) analytical system (Fig. 2). Sugars in the sake fermented by MC87-46 were separated using TLC (data not shown), and a spot with the same retention time of the isomaltose standard was identified. The mass spectrum of trimethylsilyl (TMS) derivatives from the spot indicated characteristic features of isomaltose fragmentation (Fig. S3). Glucose and panose were also identified by the same procedure (data not shown). Glucose, isomaltose, and panose were detected in sake produced by both strains (Fig. 2). Although the amount of glucose was similar in both batches of sake, levels of isomaltose and panose were 24.6- and 12.8-fold higher, respectively, in sake fermented by MC87-46 than that by K901 (Table 2). Interestingly, the concentration of isomaltose in the sake brewed with MC87-46 was 44.3 mM, indicating that the low sake meter value (−19.6) of the sake brewed with MC87-46 was caused by the high concentration of sugars, including isomaltose (Table 2), and the low concentration of ethanol (Table 1). The relative sweetness value of isomaltose is 54 compared with the 100 of glucose^27^. This suggests that the sake brewed with MC87-46 is sweet owing to the high content of isomaltose (44.3 mM). Furthermore, the time course of the concentrations of isomaltose, panose, and glucose was investigated during the fermentation process (Fig. 3A,B). The decrease in amount of glucose was similar for both MC87-46 and K901 (Fig. 3C). The initial concentration of isomaltose was 44.3 mM and was unchanged until 8 days into the fermentation of the sake by both strains (Fig. 3B). After brewing for 18 days, the isomaltose concentration in sake fermented by K901 was only 4% of the levels found in MC87-46-brewed sake, indicating that MC87-46 could not utilize isomaltose.

**Table 2:** Sugar concentrations in sake brewed with K901 and MC87-46.

	Sugar concentrations (mM)		
	Panose	Isomaltose	Glucose
K901	0.5 ± 0.03	1.8 ± 0.19	19.0 ± 2.4
MC87-46	6.4 ± 0.61*	44.3 ± 3.8*	24.0 ± 2.3

Small-scale sake was brewed for 18 days at 15 °C. Sugar concentrations were determined using a Prominence Reducing Sugar HPLC analytical system. Data are presented as mean values ± standard deviation (error bars) of four independent experiments. *p < 0.01, Student’s t test.

There is the possibility that yeast strain with poor fermentation ability accumulates high concentration of sugars, including isomaltose. To clarify this possibility, the time course of sugar metabolism using X2180 strain, which shows low fermentation activity and high isomaltase activity, was determined (Fig. S4). The decrease in amount of glucose and isomaltose by X2180 was similar to that observed with K901 (Fig. S4). These indicate that poor ethanol fermentation ability of MC87-46 does not cause the isomaltose accumulation during the fermentation process.

### Sensory evaluation and taste sensor analysis of brewed sake

We performed the sensory analysis of sake brewed with *S. cerevisiae* MC87-46 and K901 strains. Taste sensor analysis of the five gustatory attributes revealed that sourness of sake brewed with *S. cerevisiae* MC87-46 was significantly higher than that with K901 (Table 3). These correlated well with the higher acidity in the sake produced by MC87-46 than that in the sake produced by K901. Sensory evaluation showed clear differences in sweetness and sourness between sake brewed with MC87-46 and K901 (Table 3). These indicated that the isomaltose assimilation ability of *S. cerevisiae* affects the sweetness of the brewed sake. There was no significant difference in other gustatory attributes.

**Table 3:** Taste sensor analysis and sensory evaluation of sake brewed with K901 and MC87-46.

		MC87-46	K901
Taste sensor analysis	Umami	9.86 ± 0.03	10.6 ± 0.03
	Saltiness	4.09 ± 0.05	3.98 ± 0.04
	Sourness	−6.29 ± 0.05*	−16.07 ± 0.03
	Bitterness	5.04 ± 0.01	5.42 ± 0.02
	Astringency	1.00 ± 0	0.91 ± 0.03
Sensory evaluation	Sweetness	3.90 ± 1.12*	1.75 ± 0.67
	Umami	3.00 ± 0.94	2.90 ± 0.82
	Body	3.20 ± 1.01	2.50 ± 0.79
	Sourness	3.80 ± 1.20*	2.40 ± 0.76
	Bitterness	2.30 ± 0.66	2.60 ± 0.82
	Aftertaste	2.70 ± 0.85	2.71 ± 0.85

Taste intensities were evaluated on a scale of 1 (weak) to 5 (strong) for the body (1 (thin) to 5 (thick)) and sweetness (1 (dry) to 5 (sweet)). Data are presented as mean values ± standard deviation (error bars) of three independent experiments. *p < 0.01, Student’s t test.

### Assimilation of isomaltose by yeast strains

**Figure 4:**
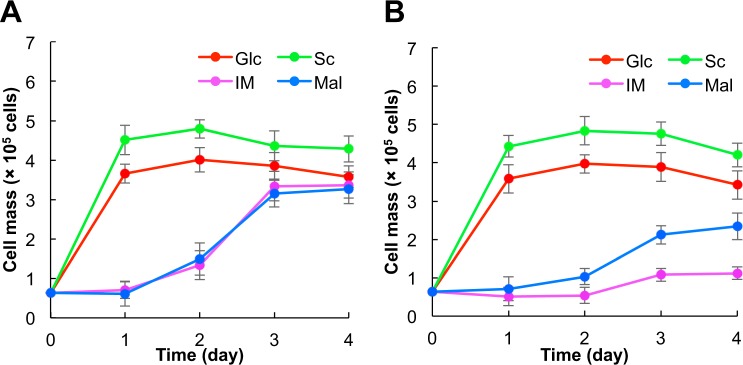
Sugar utilization of MC87-46 and K901. K901 (**A**) and MC87-46 (**B**) cultured in media containing glucose (Glc), sucrose (Sc), isomaltose (IM), and maltose (Mal) as the sole carbon source. Data are presented as mean values ± standard deviation (error bars) of four independent experiments.

Sugar availability of both MC87-46 and K901 strains was determined (Fig. 4). The growth of MC87-46 and K901 strains in YN media containing glucose or sucrose as the sole carbon source did not differ significantly (Fig. 4). Compared with the MC87-46 strain, maltose was the preferred sugar for growth of the K901 strain (Fig. 4). The MC87-46 strain barely grew in the YN medium^28^ containing 1.0% isomaltose as the sole carbon source (Fig. 4).

Isomaltase (Ima) and the isomaltose transporter (Agt1) are involved in the assimilation of isomaltose in *S. cerevisiae* S288c^28^. Five *IMA* (*IMA1-5*) and *AGT1* genes have been reported and are all located in the subtelomeric regions of different chromosomes^28^. We designed oligonucleotide primers corresponding to the *IMA1–5* and *AGT1* genes (Table S1), and each gene was sequenced. The nucleotide sequences of the *IMA3* and 4 genes of MC87-46 were identical to those of *S. cerevisiae* S288c. However, the *IMA1*, 2, 5, and *AGT1* genes of MC87-46 harboured fewer mutations compared with those in S288c and showed 99.8%, 99.7%, 94.7%, and 99.5% identities to those of S288c, respectively (Fig. S5). *IMA1-5* and *AGT1* genes of the original strain MC were identical to those of MC87-46 (data not shown), and the MC strain could not utilize isomaltose (Fig. S6). *IMA1*, *IMA2*, *IMA3*, *IMA4*, and *AGT1* genes of K901 showed 99.3%, 100%, 100%, 99.8%, and 99.7% identities to those of S288c and 99.3%, 99.7%, 100%, 99.8%, and 99.2% identities to those of MC87-46, respectively (Fig. S5). The stop codon was generated at 546 position in *IMA5* gene, indicating that the *IMA5* of K901 is a pseudogene (Fig. S5).

Heterologous expression of Ima1, the major isomaltase in *S. cerevisiae* in *Escherichia coli* expression system was previously reported^28^. Recombinant Ima1 enzymes of K901 and MC87-46 were expressed in *E. coli* and purified (Fig. S7A). To evaluate the enzymatic activity of the recombinant Ima1 enzymes, respectively, the catalytic conversions of isomaltose were analysed (Fig. S7B). The substrate was efficiently converted by each Ima1, with generation of glucose (Fig. S7B). The specific activity of Ima1 of MC87-46 was similar to that of K901 (Fig. S7C), indicating that the difference between the strains in their ability to assimilate isomaltose was not due to the enzymatic property of Ima1.

Transcriptional regulation of *IMA1-5* and *AGT1* genes was investigated by quantitative RT-PCR technique (Fig. S8). Although *IMA1* and *AGT1* of K901 strain were strongly induced by isomaltose, similar to that observed in the S288c strain^28^, none of these genes were induced in MC87-46 (Fig. S8). These results indicate that the non-induction of *IMA1* and *AGT1* genes by isomaltose are responsible for inability to assimilate isomaltose.

Cell extracts from MC87-46 and K901 incubated for 2 and 4 days with isomaltose were prepared to determine if the yeasts possessed isomaltase activity (Fig. S9). MC87-46 and K901 were pre-incubated in YM medium containing 1.0% glucose, and then transferred to YN medium with 1.0% isomaltose. Although pyruvate dehydrogenase (PDH) and glyceraldehyde-3-phosphate dehydrogenase (GAPDH) activities were similar in both strains, isomaltase activity of MC87-46 was extremely low compared with that in K901 (Fig. S9). Initial rates of isomaltose hydrolysis by isomaltase were calculated using HPLC as decreases in the isomaltose concentration. The specific activity of isomaltase in cell extracts prepared from K901 was 78.4 U/mg protein, while MC87-46 did not show any enzyme activity. Even though MC87-46 possesses five *IMA* genes, the strain could not produce active isomaltases. Isomaltase activity in MC87-46 cells correlated well with the non-induction of *IMA1* gene by isomaltose.

### Quantification of isomaltose in commercially available sake

Isomaltose concentrations in commercially available low-alcohol sake were determined (Table 4). Sake brewed with MC87-46 contained the highest isomaltose and panose levels compared with the commercial sake (Tables 2, 4). The isomaltose assimilation ability of the yeast is attractive for production of sake with new tastes.

**Table 4:** Sugar concentrations in commercial sake.

	Region	Ethanol (%)	Sugar concentrations (mM)			
			Panose	Fructose	Isomaltose	Glucose
A	Kyoto	5	4.4 ± 1.3	14.1 ± 2.1	11.1 ± 1.2	28.0 ± 3.6
B	Kyoto	10	1.6 ± 0.3	7.9 ± 1.5	4.3 ± 0.8	21.1 ± 4.3
C	Aichi	15	1.0 ± 0.3	—	5.8 ± 0.5	26.3 ± 5.2
D	Hyogo	16	1.1 ± 0.2	—	6.4 ± 0.8	35.6 ± 7.6

Commercially available sake with an ethanol concentration between 5 to 16%. Data are presented as mean values ± standard deviation (error bars) of three independent experiments.

## Discussion

In the current study, we isolated wild *S. cerevisiae* MC strain from the carnation flower and produced sake containing a high concentration of isomaltose using a cerulenin-resistant mutant MC87-46 strain. The sake meter value was −19.6 and sensory evaluation showed clear difference in sweetness of brewed sake, indicating that the sweetness of the sake fermented by MC87-46 was comparable to that of sweet dessert wines.

In Japan, the filamentous fungi *A. oryzae* has been used for over 1,000 years in the traditional food industry^29^. *A. oryzae* are generally accepted as being safe in food by the USA Food and Drug Administration (FDA) and they have been applied to produce fermented foods, including Japanese rice wine. Starch in rice is hydrolysed to glucose by *A. oryzae*, and the glucose is converted to ethanol by *S. cerevisiae* during sake brewing^1^. In addition to glucose, *A. oryzae* converts starch to various sugars including oligosaccharides such as maltose, isomaltose, panose, and others, which are also found in the sake^4,15–17^. These oligosaccharides are produced by α-amylases and α-glucosidases during sake mash making^15–17^. *Alpha*-glucosidases (EC 3.2.1.20) catalyse liberation of glucose from non-reducing ends of α-glucosides, α-linked oligosaccharides, and α-glucans^30^. Theoretically, α-glucosidase, belonging to the glycoside hydrolase family 31, is capable of catalysing transglycosylation by a retaining reaction mechanism^15,30^. *A. nidulans* α-glucosidase AgdB catalyses formation of α-1,6-glucosidic linkages in addition to hydrolysis of starch, resulting in production of isomaltose and panose from maltose^15,16^. In our study, the initial concentration of isomaltose in the sake was about 44.3 mM, indicating that isomaltose was generated by *A. oryzae* during manufacturing of rice koji.

Isomaltose concentration in sake fermented by MC87-46 was 24.6-fold more than that in the sake fermented by K901. The *S. cerevisiae* BY4741 strain can assimilate isomaltose by α-1,6-glucosidase Ima^28,29^. Five *IMA* genes (*IMA1–5*) of *S. cerevisiae* are located on different chromosomes with 65% to 99% high sequence identity (YGR287c, YIL172c, YJL216c, YJL221c, and YOL157c). Ima shows high affinity for disaccharide isomaltose and palatinose with α-1,6-linkage, and *IMA1* encodes the major isomaltase in *S. cerevisiae*^28^. In addition, growth of *S. cerevisiae* on isomaltose requires the presence of *AGT1*, which encodes an α-glucoside transporter^28^. These genes were not induced by isomaltose in MC87-46, and the strain did not possess isomaltase activity. These results indicated that MC87-46 could not utilize isomaltose, resulting in a high concentration of isomaltose in sake fermented with MC87-46.

Isomalto-oligosaccharides (IMOs), including isomaltose, are a heterogeneous group of glycosyl saccharides with an α-1,6-linkage. These oligosaccharides are used in the food, pharmaceuticals, and cosmetics industries due to their unique prebiotic properties^30–34^. IMOs competitively inhibited rat small intestinal α-glucosidases, such as sucrase, maltase, and glucoamylase, and reduced the rate of hydrolysis of sucrose and other α-glucosyl saccharides, including maltose, dextrin, or soluble starch^31^. These oligosaccharides selectively promote the growth of *Bifidobacterium* in a dose-dependent manner in the human intestine^31–33^. Beside their prebiotic effect, IMOs have beneficial effects on blood cholesterol regulation, immunomodulation, and prevention of various diseases^34^. The MC87-46 strain would be useful for preparing traditional Japanese sake with additional nutrient functions.

In summary, we used a *S. cerevisiae* MC87-46 isolated from the carnation flower, with subsequent introduction of cerulenin-resistance, to produce sake. The brewed sake was sweet due to high levels of the oligosaccharide isomaltose, which was 24.6-fold more than that found in the commercial sake that we tested. These findings suggested that MC87-46 could be useful for commercial application in Japanese sake production, providing a new taste and nutrient functions.

## Methods

### Chemicals

Isomaltose (6-*O*-α-d-glucopyranosyl-d-glucopyranose) was purchased from Tokyo Chemical Industry (Tokyo, Japan). Panose (6-*O*-α-glucopyranosyl-maltose) was obtained from Hayashibara (Okayama, Japan). Glucose, maltose, raffinose, and sucrose were purchased from Wako Pure Chemical Industries (Osaka, Japan).

### Isolation and identification of wild *S. cerevisiae*

*S. cerevisiae* was isolated from the carnation flower (*Dianthus caryophyllus L*) grown in an experimental farm at Meijo University with the use of selective medium, as previously described^35^. Flowers were preserved in liquid Yeast Nitrogen Base medium (6.7 g/L) with raffinose (6.6 g/L) and ethanol (80 mL/L), then incubated at 28 °C for 72 h. Raffinose was used for the isolation of *S. cerevisiae* because *Saccharomyces* species are capable of utilizing raffinose for growth. Aliquots of 100 μL from the culture were plated onto YPG agar (10 g/L yeast extract, 10 g/L peptone, 10 g/L glucose, 20 g/L agar) supplemented with chloramphenicol (30 mg/L) to inhibit bacterial growth. Yeast colonies were isolated after incubation at 26 °C for 3–5 days.

Sequencing of the ITS1/ITS2 region of the *18S rRNA* gene was used to identify the strain of *S. cerevisiae*. DNA was extracted from the yeast cells as described^23,36^ and DNA fragments encoding the *18S rRNA* gene were amplified with gene-specific primer sets (Table S1). The wild *S. cerevisiae* MC strain isolated from the carnation flower was used to brew sake in this study.

### Selection of cerulenin-resistant *S. cerevisiae* strain

A cerulenin-resistant *S. cerevisiae* strain was generated using a procedure previously described^24^. Briefly, the wild *S. cerevisiae* MC strain was cultured in 5 mL of YPG medium at 30 °C for 24 h. The culture was centrifuged at 3,000 × *g* for 5 min, and the cell pellet was washed with sterile water. The yeast cells were suspended in 5 mL of 0.2 M phosphate buffer (pH 8.0), 0.25 mL of 40% glucose, and 0.2 mL of ethyl methanesulfonate (EMS), and incubated at 30 °C for 1 h. The mutagenized yeast cells were washed with sterile water, plated on YPG agar medium containing 50 μM cerulenin and incubated at 30 °C for 7 days. The strain that had the largest colony diameter was selected as the cerulenin-resistant MC87 strain.

### Selection of an ethanol-resistant strain

The cerulenin-resistant MC87 strain was cultured in YPG medium at 30 °C for 24 h, centrifuged, and resuspended in acetate buffer (pH 4.2). The cell suspension was centrifuged at 3,000 × *g* for 5 min, and the supernatant was discarded. The cell pellet was again resuspended in acetate buffer (pH 4.2) containing 18% (v/v) ethanol and incubated for 4 h. The cell suspension (100 μL) was plated on YM agar (3.0 g/L yeast extract, 3.0 g/L maltose extract, 10.0 g/L glucose, 5.0 g/L peptone, and 20.0 g/L agar). After 2 days of incubation, yeast colonies were picked and then resuspended in acetate buffer (pH 4.2) containing 20% (v/v) ethanol and incubated at 15 °C for 7 days^25^. The cell suspension was plated on YM agar and incubated at 30 °C for 2 days. One colony that grew was selected as the ethanol-resistant MC87-46 strain.

The *S. cerevisiae* MC87-46 strain has been deposited as NBRC 113767 in the International Patent Organism Depositary, National Institute of Technology and Evaluation (NITE; Tokyo, Japan).

### Ethanol tolerance test

The yeast strains were cultured in YPG medium at 30 °C for 24 h with shaking. The collected cells were adjusted to optical density value at 660 nm (OD_660_) = 1.0 and were incubated in 22% ethanol solution at 30 °C^26^. The number of surviving cells was confirmed by counting colony-forming units on YPD plates.

### Small sake brewing

Small-scale sake brewing was carried out as described previously with some modifications^3^. *S. cerevisiae* MC87-46 and *S. cerevisiae* Kyoukai No. 901 (K901) obtained from the Brewing Society of Japan were cultured in YPG medium at 30 °C for 2 days and then harvested by centrifugation at 200 × *g*. The small-scale sake brewing test included 10 mL of pre-incubated yeast culture (1 × 10^6^ cells/mL), 40 g of dried rice (Tokushima Seikiku, Osaka, Japan), 10 g of rice koji (Tokushima Seikiku), and 90 mL of water mixed in 250 mL glass bottles. Fermentation proceeded for 18 days at 15 °C. The weight of the sake mash was measured to monitor carbon dioxide evolution. Prepared sake (100 mL) was separated by centrifugation at 8,000 × *g* for 5 min, and the resulting supernatant was filtered through a 0.22-μm filter.

### Analytical methods

Sake meter value, sake acidity, and amino acid levels were measured as previously described^6^. Organic acids were quantified using HPLC with a Shim-pack SCR-102H column (Shimadzu, Kyoto, Japan). Aroma components were quantified using headspace GC with a J & W DB-WAX capillary column (30 m × 0.32 mm internal diameter × 0.50 μm film thickness; Agilent Technologies, Santa Clara, California, USA). Ethanol concentration was measured using an ethanol analyser (RIKEN KEIKI, Tokyo, Japan). Sugars were determined by monitoring post-column derivative reducing sugars separated using a Prominence reducing-sugar HPLC analytical system (Shimadzu) equipped with a fluorescence detector. Sake fermented with MC87-46 and K901 was separated on a Shim-pack 4.0 × 250-mm ISA-07/S2504 column (Shimadzu) with a linear gradient of 0.1 M potassium borate buffer (pH 8.0) and 0.4 M potassium borate buffer (pH 9.0) for 140 min at a flow rate of 0.6 mL/min^37,38^. Sugars were also lyophilized, trimethylsilylated, and analysed using a GCMS-QP2010 (Shimadzu) equipped with a J & W DB-5MS capillary column (30 m × 0.25 mm internal diameter × 0.25 μm film thickness; Agilent Technologies)^39^.

### Sensory evaluation and taste sensor analysis

The taste of sake was evaluated by ten students, belonging to Japanese sake study club of Meijo University, experienced in sensory evaluation of sake. The samples were evaluated for six attributes (sweetness, umami, sourness, bitterness, aftertaste, body) and scored on a scale from 1 (weak) to 5 (strong) for body (1 (thin) - 5 (thick)) and sweetness (1 (dry) - 5 (sweet))^26^.

A taste sensing apparatus (SA402B, Intelligent Sensor Technology Inc., Kanagawa, Japan) was used for the taste sensor analysis, according to the manufacturer’s instructions.

### Ethical permission

All experiments were performed according relevant guidelines and regulations of Meijo University. The Research Commission of the Faculty of Agriculture at Meijo University approved the procedures and methodologies related to the sensory evaluation of Japanese sake brewed with K901 and MC87-46 strains. All participants of the sensory evaluation tests signed an informed consent form on the type of the research being conducted; they agreed to sensory evaluation of the Japanese sake that was produced as described in the Methods section under “Small sake brewing.”

### Yeast cell growth using various sugars

MC87-46 and K901 (1 × 10^5^ cells) were inoculated in YN medium supplemented with glucose, sucrose, maltose, or isomaltose (1.0% each) at 30 °C with shaking at 120 rpm^28^. Cell growth was monitored by optical density at 600 nm.

### Cloning of IMA1–5 and AGT1 genes

Total RNA was prepared from disrupted yeast cells using the RNeasy Mini Kit (Qiagen, Venlo, The Netherlands), according to the manufacturer’s instructions. Single-stranded cDNA was synthesized from total RNA extracted, and cDNA fragments encoding the full-length *IMA1-5* and *AGT1* genes were prepared by PCR using gene-specific primer sets (Table S1). The PCR product was cloned, and the nucleotide sequence was determined using an automated DNA sequencer (CEQ2000, Beckman Coulter, Brea, CA, USA).

### Preparation of recombinant proteins

The PCR product of *IMA1* gene was purified, and then ligated into pET21a (Novagen, Darmstadt, Germany), which had been digested with the restriction enzyme *Bam*HI. Plasmid for producing recombinant Ima1 of K901 was constructed by the same strategy.

The plasmids were introduced into *E. coli* BL21-CodonPlus(DE3) (Novagen), cultured in LB containing 100 μg/L sodium ampicillin for 12 h, and then a portion (3 mL) was subjected to rotary shaking at 120 rpm in 200 mL of LB containing 100 μg/L sodium ampicillin at 30 °C. Isopropyl-thio-β- d-galactoside (0.2 mM) was added to the medium when the OD_600_ reached 1.0, and the flasks were rotary-shaken for 8 h at 120 rpm and 30 °C. The *E. coli* cells were harvested, suspended in 50 mL of 20 mM sodium phosphate buffer (pH 7.0) containing 1 mM EDTA and 0.1 mM PMSF, and disrupted by sonication. The sonicate was centrifuged at 6,000 × *g* for 10 min, and the soluble fraction was separated by centrifugation at 20,000 × *g* for 15 min. These fractions were passed through a column containing nickel-nitrilotriacetic acid agarose (ϕ5 × 20 mm, Qiagen) that was washed with 10 mL of 20 mM sodium phosphate (pH 7.0) containing 20 mM imidazole. Proteins were eluted with the same buffer containing 300 mM imidazole. All procedures were carried out at temperatures below 4 °C. The resultant fraction was dialyzed against 20 mM sodium phosphate (pH 7.0) and analysed.

### Enzyme assays

MC87-46 and K901 cells (OD_600_ nm = 1.0) grown in 10 mL of YN medium containing 1.0% glucose (w/v) were collected by centrifugation (2,000 × *g*, 5 min). The cell pellets were washed twice with 1.0 mL of Milli Q water and transferred to a test tube containing 10 mL of YN medium with 1.0% isomaltose (w/v). After incubation at 25 °C with shaking at 120 rpm, cells were collected and resuspended in 700 μL of 0.1 M potassium phosphate buffer (pH 6.8) containing 1 mM phenylmethylsulfonyl fluoride (PMSF), 1 mM dithiothreitol (DTT), and an equal volume of glass beads (0.4–0.5 mm diameter). The yeast cells were disrupted by vortexing four times for 30 s each. The mixture was centrifuged for 5 min at 15,000 × *g*, and the supernatant was used for enzymatic assays. Isomaltase activity was assayed in 0.5 mL reaction mixtures containing 50 mM sodium phosphate (pH 6.8), 1.0% isomaltose and cell extracts at a final protein concentration of 0.5 mg/mL or purified recombinant IMAs. Reactions were incubated at 30 °C for 3 h, and proteins were removed from the reaction using a Nanosep® Centrifugal Device (Pall Corporation, Port Washington, NY, USA)^40^. Flow-through fractions were boiled at 100 °C for 5 min, and the glucose produced from isomaltose by IMA was determined using the reducing-sugar HPLC analytical system (Shimadzu) and TLC Silica gel 60 plates (Merck-Millipore) using n-butanol:water:pyridine:toluene (10:6:6:1) visualized by staining with Aniline-phthalic acid solution (2 mL of aniline containing 3.25 g of phthalic acid). Standard curves were prepared based on solutions containing various concentrations of glucose^39^. One unit of isomaltase activity was defined as the amount of enzyme required to produce 1 µmol of reducing sugar (glucose equivalents) per minute. Protein concentrations in cell extracts were assayed using Bradford Protein Assays (Bio-Rad Laboratories, Hercules, CA, USA) with bovine serum albumin as the standard.

PDH and GAPDH activities were assayed using pyruvate dehydrogenase activity colorimetric assay and GAPDH activity assay kits (BioVision, Mountain View, CA, USA), respectively.

### Quantitative RT-PCR analysis of IMA1–5 and AGT1 genes

After a 2-day pre-incubation in YN medium at 30 °C with shaking at 120 rpm, isomaltose (final concentration at 2.0%) was added to the cultures and the cells were incubated for an additional 12 h. Total RNA was extracted from the cells grown in the absence or presence of isomaltose using the RNeasy Mini Kit (Qiagen). Single-stranded cDNA was then synthesized from the total RNA. Quantitative RT-PCR was performed using gene-specific primer sets (Table S1) designed by Teste *et al*.^28^. Gene expression was normalized to the expression of the *ALG9* gene^28^. The expression of each gene in the presence of isomaltose is shown relative to its expression in the absence of the substrate.

## Supplementary information

Supplemental_information

## Data Availability

All data generated or analysed during this study are included in this published article and supplementary information.
