# Supplementary material for: Ability of Saccharomyces cerevisiae MC87-46 to assimilate isomaltose and its effects on sake taste
Source: Sci Rep. 2019 Sep 26;9:13908. doi: 10.1038/s41598-019-50384-w (PMC6763438; doi:10.1038/s41598-019-50384-w)

**Supplemental information**

**Ability of *Saccharomyces cerevisiae* MC87-46 to assimilate isomaltose and its effects on sake taste**

**Seitaro Tsutsumi^1,^**^§^**, Mai Mochizuki^1,^**^§^**, Kiyota Sakai^1,^**^§^**, Akane Ieda^1^, Reiji Ohara^1^, Shun Mitsui^2^, Akitoshi Ito^2^, Tatsuya Hirano^1^, Motoyuki Shimizu^1,^*, Masashi Kato^1,^***

*^1^ Faculty of Agriculture, Meijo University, 1-501, Shiogamaguchi, Tenpaku-ku, Nagoya, Aichi, 468-8502, Japan*

*^2^ Food Research Centre, Aichi Centre for Industry and Science Technology, 2-1-1 Shimpukuji-cho, Nishi-ku, Nagoya, Aichi, 451-0083, Japan*

**Corresponding author. Tel/Fax: +81-52-838-2443.*

*E-mail: moshimi@meijo-u.ac.jp (M. Shimizu), mkato@meijo-u.ac.jp (M. Kato).*

^§^These authors contributed equally to this work.

**Supplementary Table S1.** Oligonucleotide primers used in this study

| Primer | Gene | Nucleotide sequence |
| --- | --- | --- |
| Sequence for the ITS1/ITS2 region of 18S rRNA gene | | |
| *ITS-*Fw | *ITS* | 5'-GTAACAAGGTTTCCGT-3' |
| *ITS*-Rv |  | 5'-CGTTCTTCATCGATG-3' |
| Cloning of *IMA1-5* and *AGT1* genes | | |
| *IMA1*-Fw | *IMA1* | 5'-ATGACTATTTCTTCTGCACA-3' |
| *IMA1*-Rv |  | 5'-TCATTCGCTGATATATATTC-3' |
| *IMA2*-Fw | *IMA2* | 5'-ATGACTATTTCTTCTGCACA-3' |
| *IMA2*-Rv |  | 5'-TCATTCAGATATGTAAATTC-3' |
| *IMA3*-Fw | *IMA3* | 5'-ATGACTATTTCTTCTGCACA-3' |
| *IMA3*-Rv |  | 5'-TCATTCAGATATGTAAATTC-3' |
| *IMA4*-Fw | *IMA4* | 5'-ATGACTATTTCTTCTGCACA-3' |
| *IMA4*-Rv |  | 5'-TCATTCAGATATGTAAATTC-3' |
| *IMA5*-Fw | *IMA5* | 5'-ATGACGATCATCCATAATCC-3' |
| *IMA5*-Rv |  | 5'-TTACTTCAACAAGTAAAGTC-3' |
| *AGT1*-Fw | *AGT1* | 5'-ATGAAAAATATCATTTCATT-3' |
| *AGT1*-Rv |  | 5'-TTAACATTTATCAGCTGCAT-3' |
| Real time PCR | | |
| rt*IMA1*-Fw | *IMA1* | 5'-CGATGCCATTTGGATCTCACCATTC-3' |
| rt*IMA1*-Rv |  | 5'-ACCAGTCACGCTTTGGATTAGTCTTC-3' |
| rt*IMA2*-Fw | *IMA2* | 5'-GACTTAGTCATCAACCATTGCTCG-3' |
| rt*IMA2*-Rv |  | 5'-ACAGTCCTCATTCTCCCAATTCAA-3' |
| rt*IMA3*-Fw | *IMA3* | 5'-GACTTAGTCATCAACCATTGCTCC-3' |
| rt*IMA3*-Rv |  | 5'’-GCAGTCTTCGTTCTCCCAGTTTAG-3' |
| rt*IMA4*-Fw | *IMA4* | 5'-GACTTAGTCATCAACCATTGCTCC-3' |
| rt*IMA4*-Rv |  | 5'-GCAGTCTTCGTTCTCCCAGTTTAG-3' |
| rt*IMA5*-Fw | *IMA5* | 5'-ACAATGACGGATGGGGTGATTTAGC-3' |
| rt*IMA5*-Rv |  | 5'-CAACAATAACCTTGATACCTCTCTTATGAGC-3' |
| rt*AGT1*-Fw | *AGT1* | 5'-GTCTTGGGTTAGCGGGTACA-3' |
| rt*AGT1*-Rv |  | 5'-CCATTACTAGCGCTGCTTCC-3' |
| rt*ALG9*-Fw | *ALG9* | 5'-CACGGATAGTGGCTTTGGTGAACAATTAC-3' |
| rt*ALG9*-Rv |  | 5'-TATGATTATCTGGCAGCAGGAAAGAACTTGGG-3' |
| Plasmids for recombinant protein production | | |
| rIma1-Fw | *IMA1* | 5'-GGTCGCGGATCCGAATTCGAGCTCATGACTATTTCTTCTGCACA-3' |
| rIma1-Rv |  | 5'-CGCAAGCTTGTCGACGGAGCTCGCTTCGCTGATATATATTCTTC-3' |

**Supplementary Figure S1.** Ethyl caproate production of MC and MC87 strains.

Small-scale sake was brewed for 18 days at 15 °C. Data are presented as mean values ± standard deviation (error bars) of three independent experiments.

**Supplementary Figure S2.** Ethanol tolerance of MC87 and MC87-46 strains.

Yeast cells were cultured in YPD medium at 30 °C for 24 h with shaking. Suspensions of MC87, and MC87-46 cells adjusted to OD_660_ **=** 1.0 were exposed to 22% ethanol at 30 °C. Survival rates of yeast cells were evaluated by counting colony-forming units on YPD plates. Data are presented as mean values ± standard deviation (error bars) of three independent experiments.

**Supplementary Figure S3.** Identification of isomaltose in sake fermented with MC87-46.

(A) Total ion chromatogram of isomaltose standard and isomaltose in sake fermented with MC87-46. Peak at retention time of 62.72 min and 63.38 min on GC is TMS-isomaltose. (B) Mass spectrum of isomaltose in sake fermented by MC87-46. Mass spectrum was obtained from GC peak at retention time of 62.72 min (TMS-isomaltose).

A

MC87-46

IM standard

B


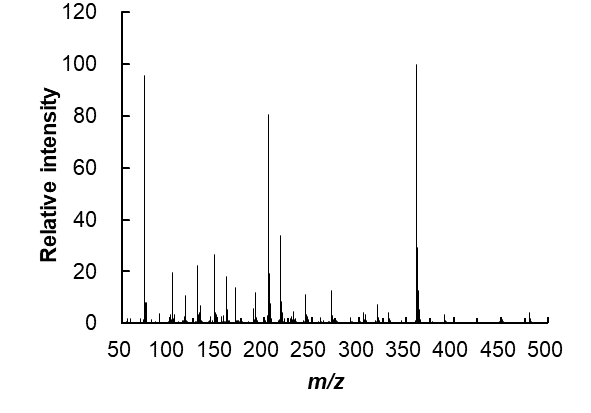


73

103

129

147

169

204

243

271

319

361

390

450

480

**Supplementary Figure S4.** Time course of sugar concentration in YN medium with glucose and isomaltose.

X2180 (A), K901 (B) and MC87-46 (C) strains were cultured in YN medium containing 50 mM glucose and 50 mM isomaltose with shaking at 120 rpm at 30 °C. Data are presented as mean values ± standard deviation (error bars) of three independent experiments.

A B C

**
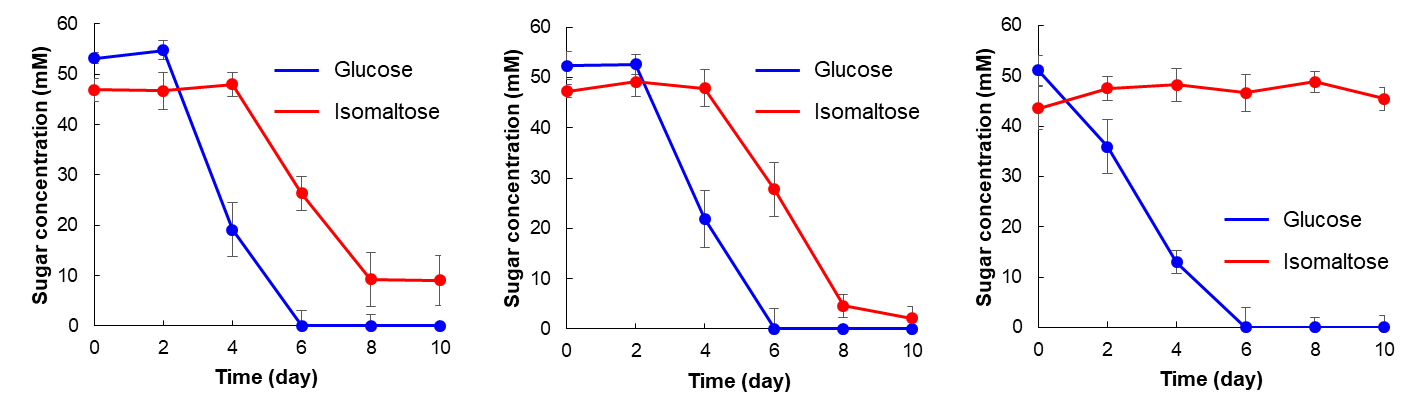
**

**Supplementary Figure S5.** Multiple amino acid sequence alignments of Ima1 (A), Ima2 (B), Ima4 (C), Ima5 (D), and Agt1 (E) from *S. cerevisiae* S288c, MC87-46, and K901 strains.

Amino acid sequences were aligned using ClustalW software. Compared with each protein of S288c, different amino acids are highlighted. Dots and colons indicate conserved amino acids with substitutions. Dashes indicate gaps.

A


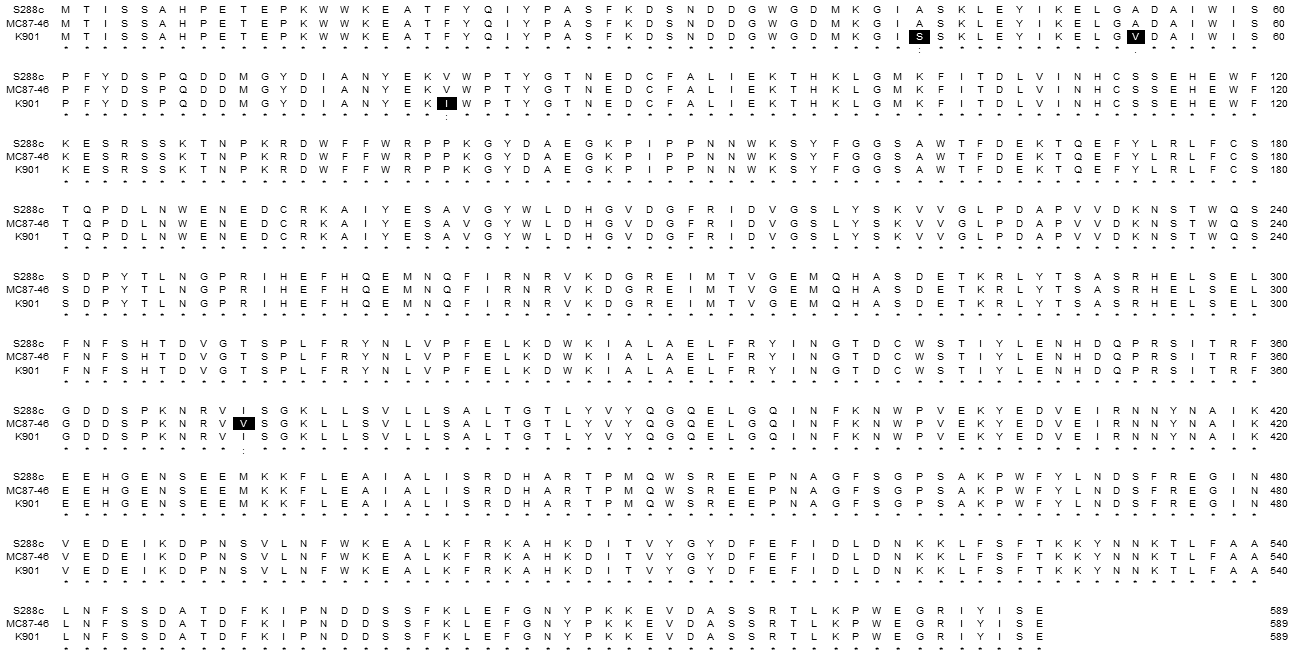


B


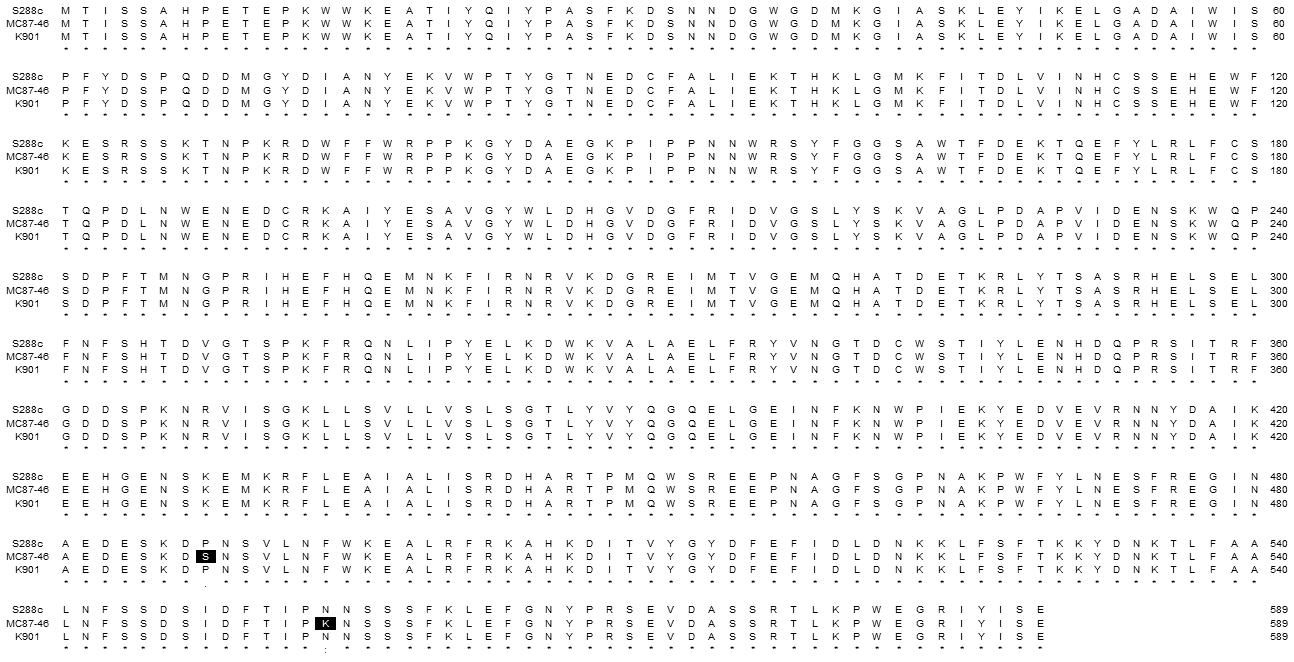


C


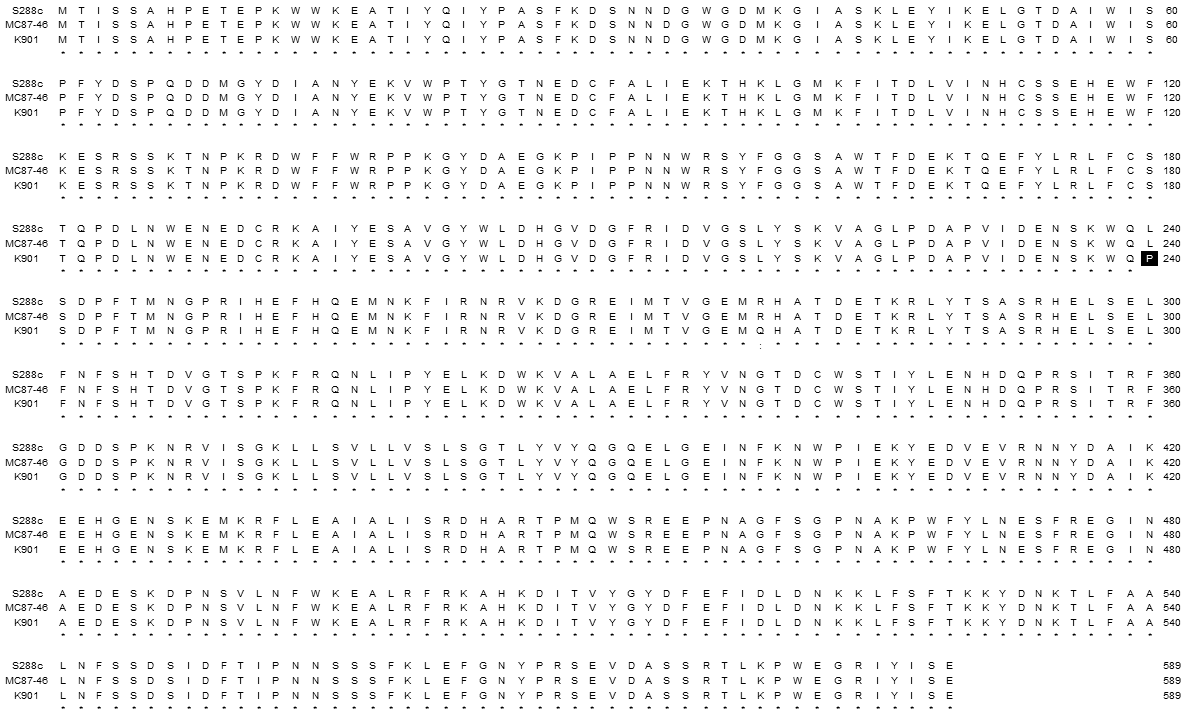


D


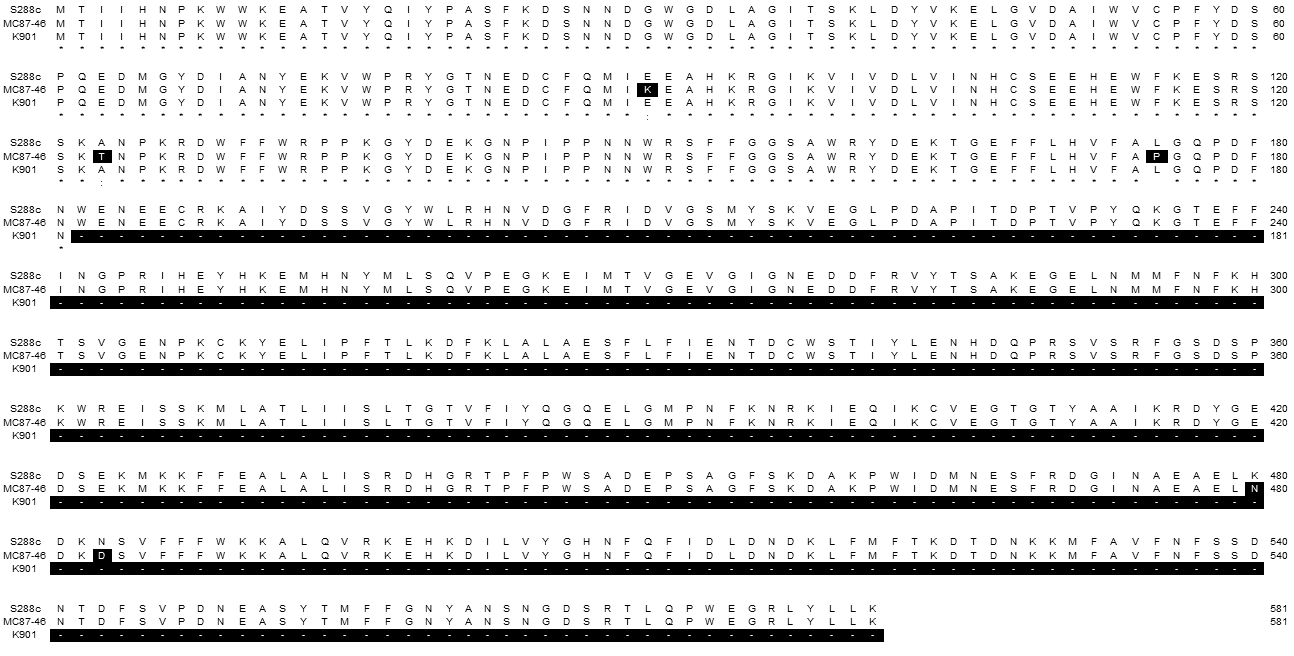


E


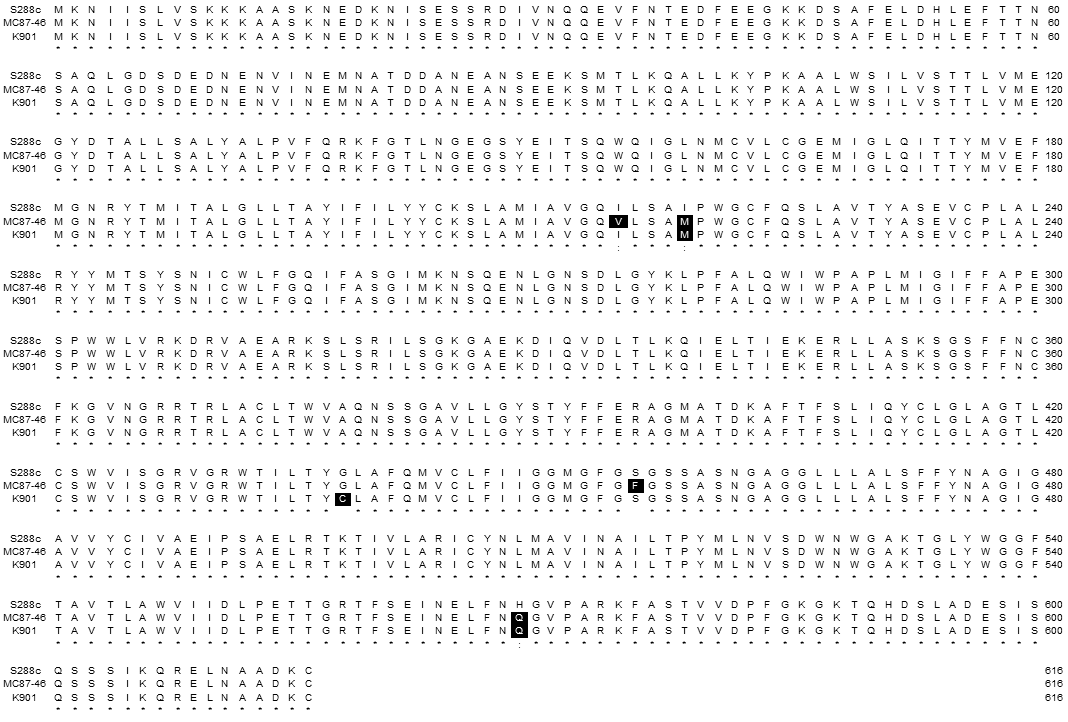


**Supplementary Figure S6.** Sugar utilization of X2180 and MC strains.

X2180 (A) and MC (B) strains cultured in media containing glucose (Glc) and isomaltose (IM) as the sole carbon source. Data are presented as mean values ± standard deviation (error bars) of three independent experiments.

A B

**Supplementary Figure S7.** SDS-PAGE analysis and isomaltase activity of recombinant Ima1 proteins of MC87-46 and K901.

(A) SDS-PAGE analysis of purified Ima1 proteins of MC87-46 and K901. *M*, protein molecular-mass markers; *lane* 1, recombinant Ima1 of MC87-46; *lane* 2, recombinant Ima1 of K901. (B) TLC analysis of soluble products from isomaltose in the reaction mixtures containing purified Ima1 proteins of MC87-46 or K901. *Std.*, isomaltose (IM) and glucose (Glc) standards; *lane* 1, Soluble products by Ima1 of MC87-46; *lane* 2, Soluble products by Ima1 of K901. (C) Specific activity of purified Ima1 proteins of MC87-46 and K901. Ima1 (1.0 μM) was incubated with 10 mM isomaltose in 50 mM sodium phosphate buffer (pH 6.8) at 30°C for 2 h. Data are presented as means ± standard deviation (error bars) of four experiments.

A B C

**Supplementary Figure S8.** Gene expression profiles of *IMA1-5* and *AGT1* in response to exogenous isomaltose.

MC87-46 and K901 (1 × 10^5^ cells) were incubated in YN medium for 2 days. After a 2-day incubation, isomaltose (final concentration at 2 %) was added to the cultures. The cells were incubated for an additional 12 h. The abundance of amplified cDNA fragments of *IMA1*, *IMA2*, *IMA3*, *IMA4*, *IMA5*, and *AGT1* transcripts was normalized by comparing with that of *ALG9*. The relative normalized expression of each gene in yeasts upon a 12-h exposure to isomaltose is shown as a ratio to the normalized expression in the absence of isomaltose. Data are presented as mean values ± standard deviation (error bars) of four independent experiments.


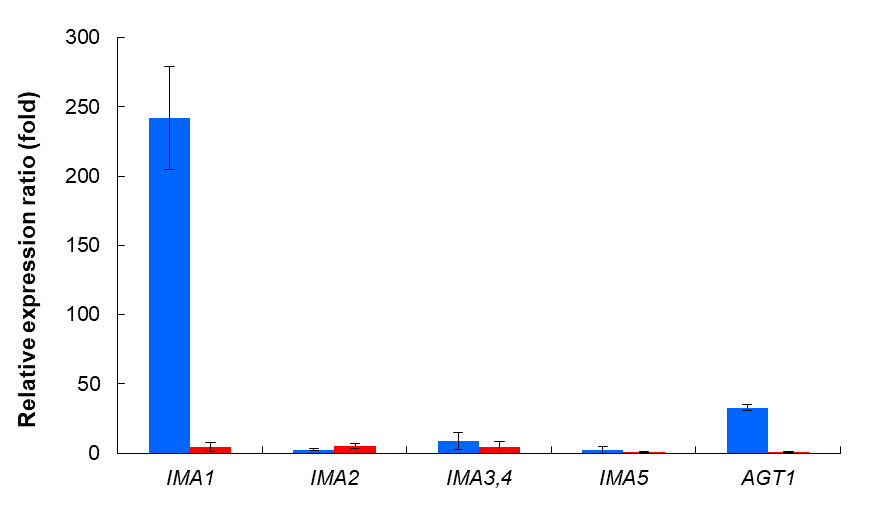


K901

MC87-46

**Supplementary Figure S9.** Isomaltase, PDH and GAPDH activities of MC87-46 and K901 cell extracts.

(A) TLC analysis of soluble products in the reaction mixtures of MC87-46 and K901 cell extracts (final protein concentration of 0.5 mg/mL) cultured with 1.0% isomaltose for 2 and 4 days. *Std.*, isomaltose (IM) and glucose (Glc) standards; *lane* 1, K901 cell extracts cultured for 2 days; *lane* 2, K901 cell extracts cultured for 4 days; *lane* 3, MC87-46 cell extracts cultured for 2 days; *lane* 4, MC87-46 cell extracts cultured for 4 days. (B) PDH and (C) GAPDH activities of MC87-46 and K901 cell extracts. Data are presented as mean values ± standard deviation (error bars) of three independent experiments.

A B C


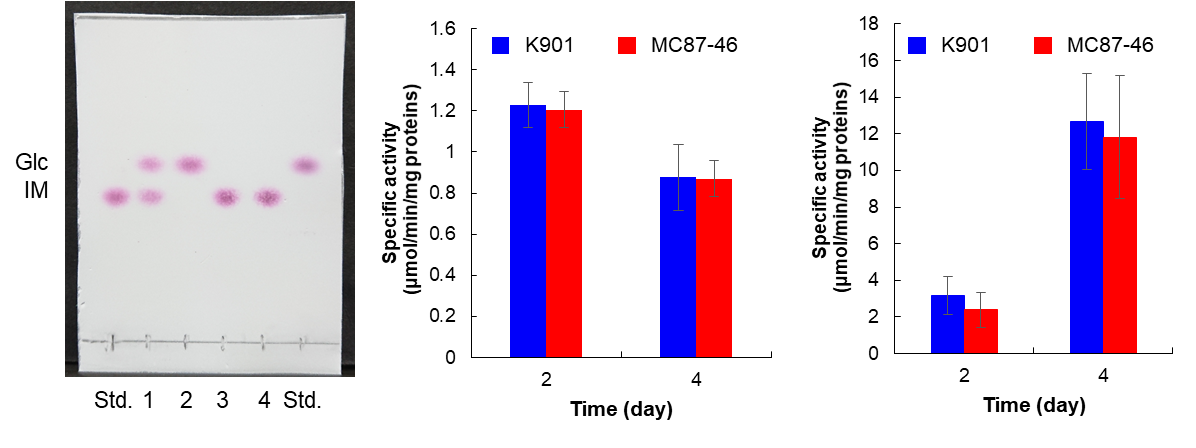

Supplement: Supplementary file 1 — Supplemental_information [file 41598_2019_50384_MOESM1_ESM.docx]
